# Supplementary figures and images for: A specific role for importin-5 and NASP in the import and nuclear hand-off of monomeric H3
Source: eLife. 2022 Sep 6;11:e81755. doi: 10.7554/eLife.81755 (PMC9560165; doi:10.7554/eLife.81755)

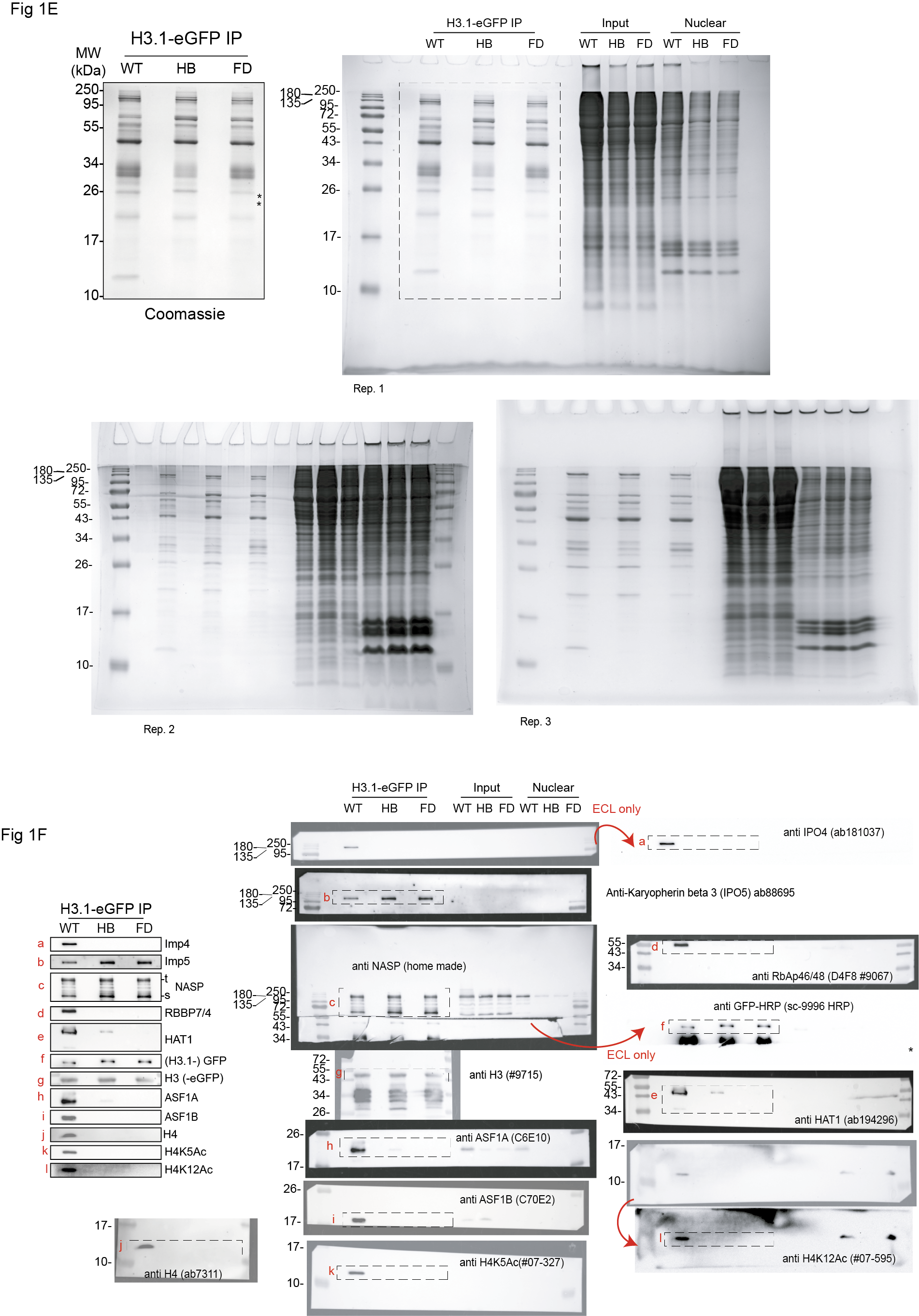


NEB Color Prestained Protein Standard, Broad Range (10-250 kDa)


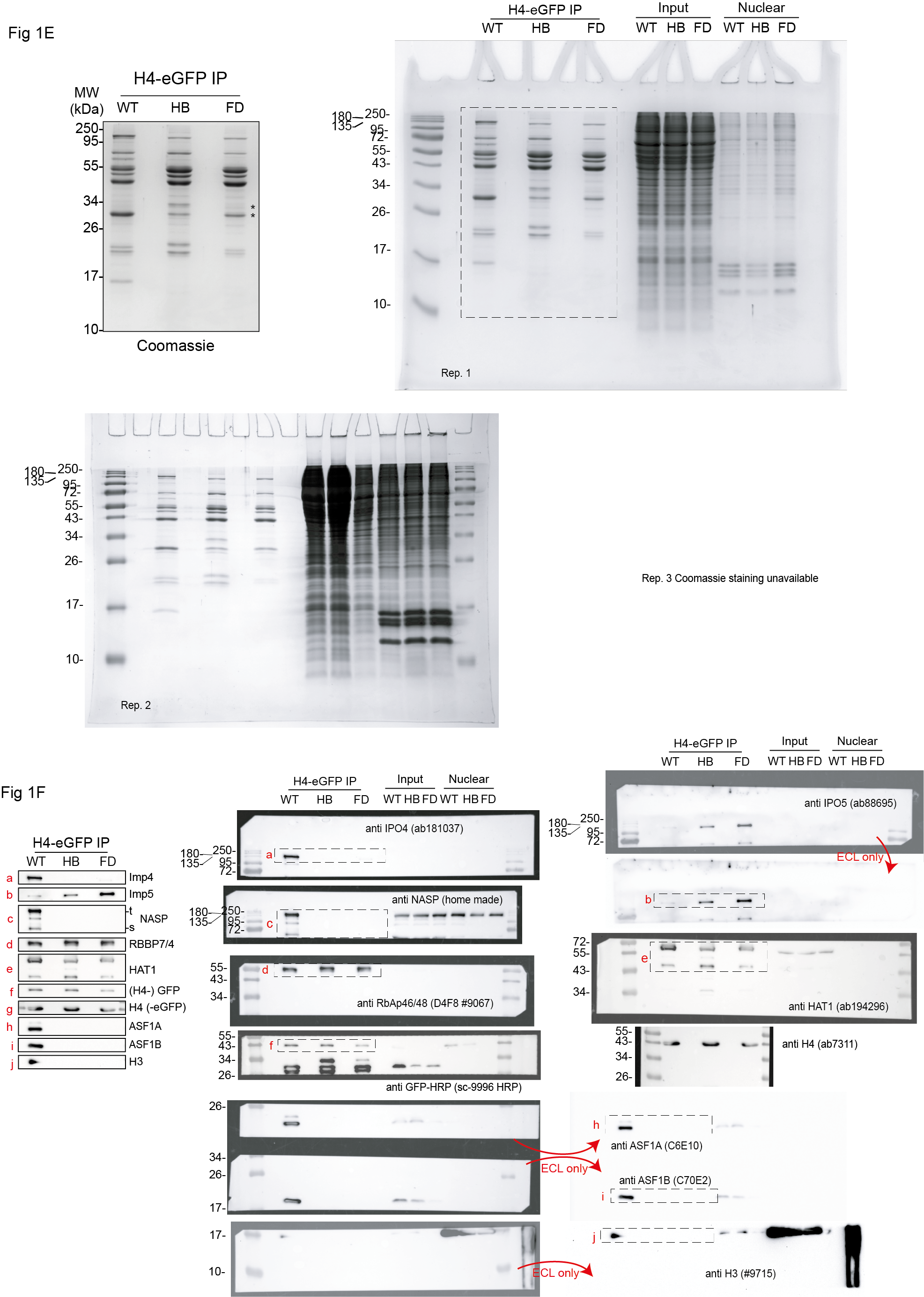


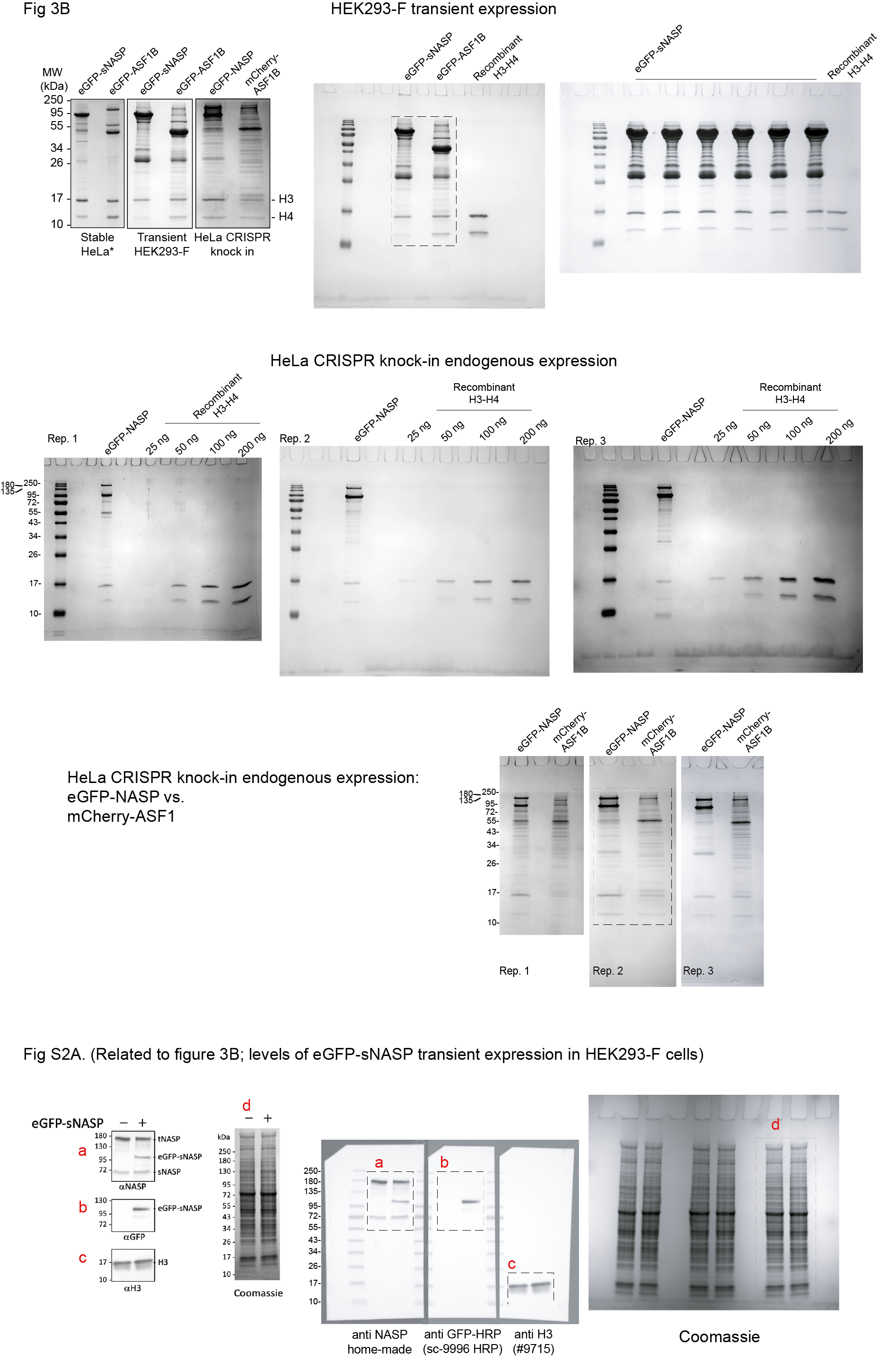


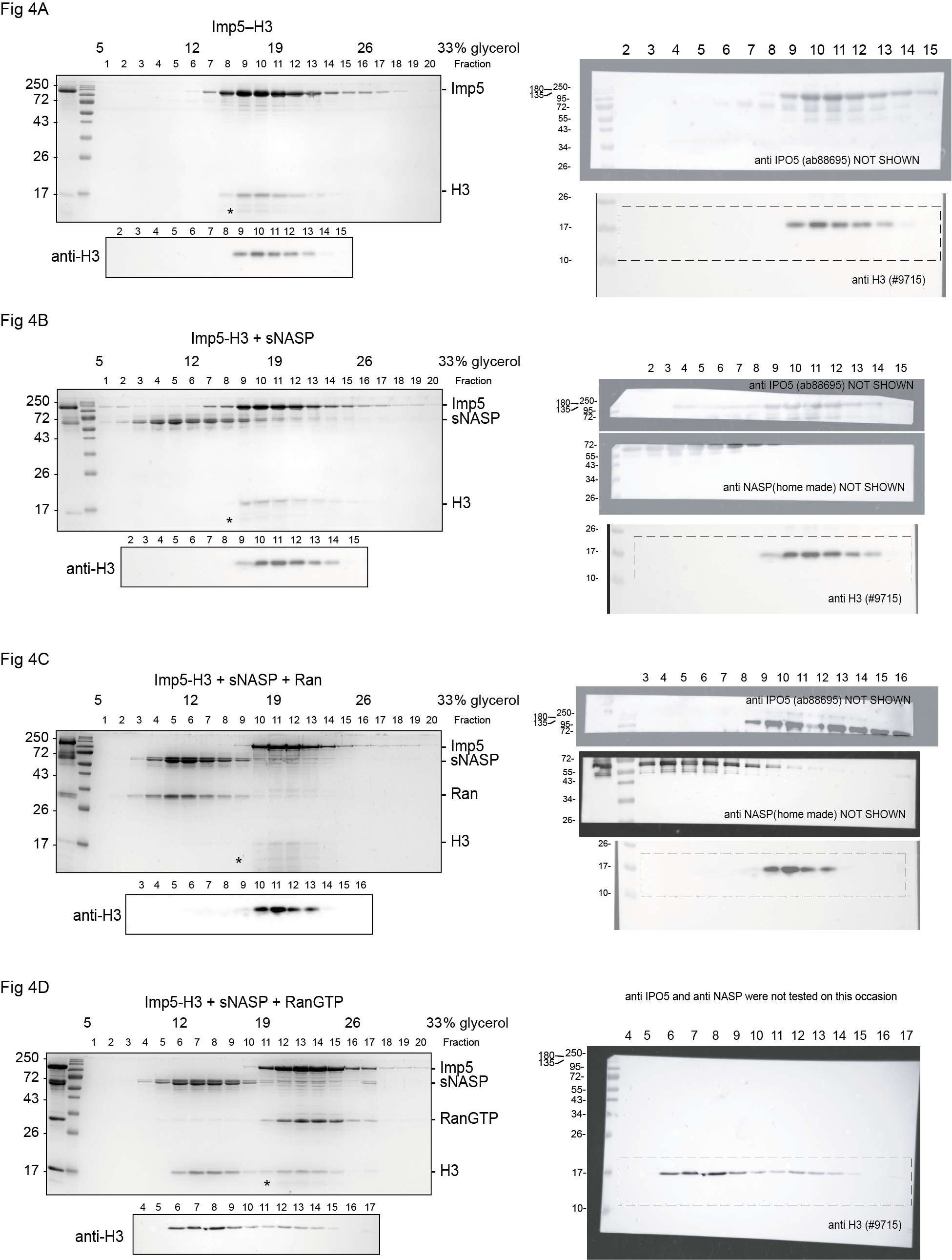


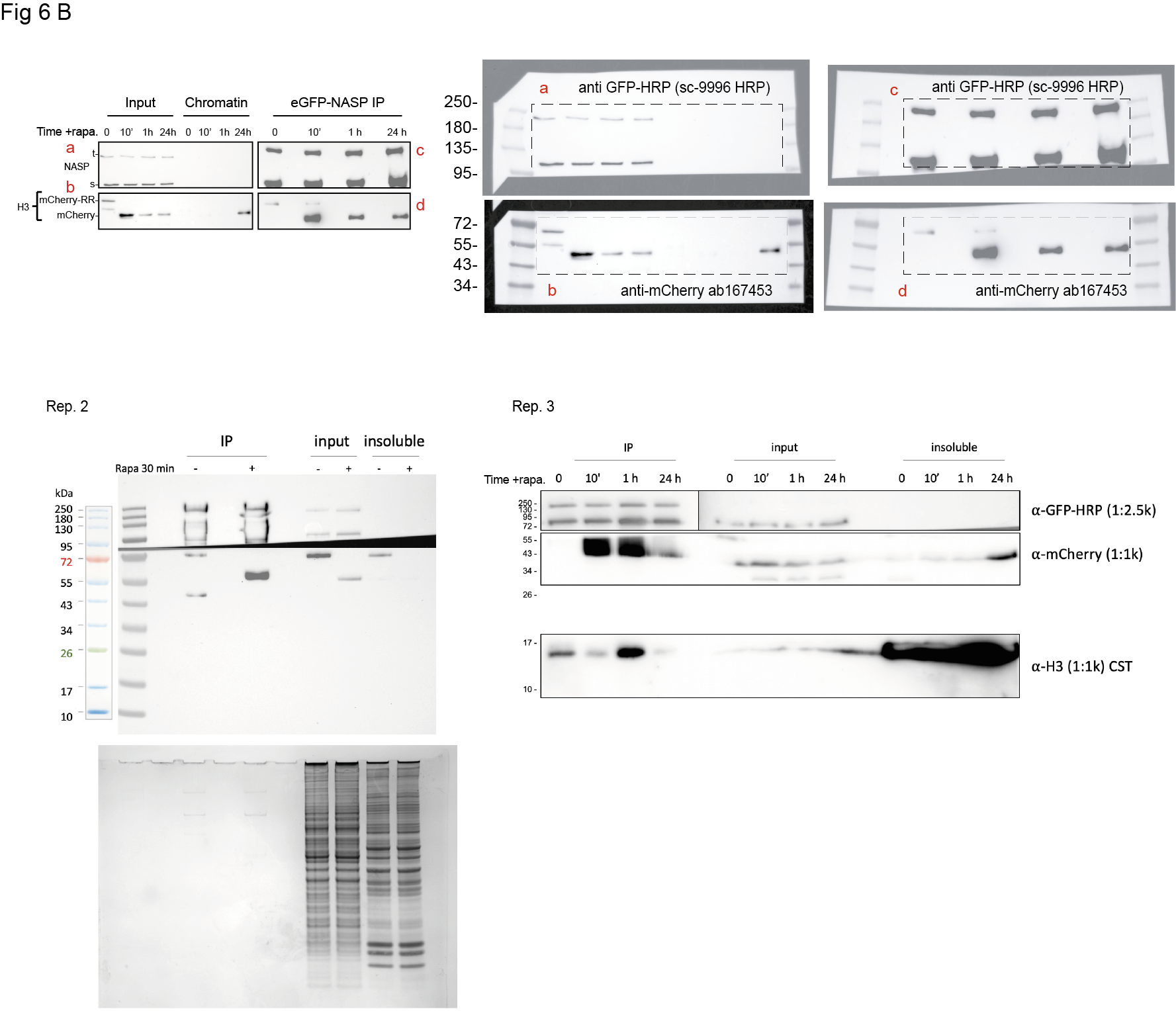

Supplement: Source data 1. [file elife-81755-data1.docx]
