# Supplementary material for: A specific role for importin-5 and NASP in the import and nuclear hand-off of monomeric H3
Source: eLife. 2022 Sep 6;11:e81755. doi: 10.7554/eLife.81755 (PMC9560165; doi:10.7554/eLife.81755)
Supplement: Transparent reporting form [file elife-81755-transrepform1.pdf]

eLIFE
1st Floor
24 Hills Road
Cambridge CB2 1JP, UK
P 01223 855340
W elifesciences.org
T @elife

eLife's transparent reporting form

We encourage authors to provide detailed information *within their submission* to facilitate the interpretation and replication of experiments. Authors can upload supporting documentation to indicate the use of appropriate reporting guidelines for health-related research (see EQUATOR Network), life science research (see the BioSharing Information Resource), or the ARRIVE guidelines for reporting work involving animal research. Where applicable, authors should refer to any relevant reporting standards documents in this form.

If you have any questions, please consult our Journal Policies and/or contact us: editorial@elifesciences.org.

Sample-size estimation

- You should state whether an appropriate sample size was computed when the study was being designed
- You should state the statistical method of sample size computation and any required assumptions
- If no explicit power analysis was used, you should describe how you decided what sample (replicate) size (number) to use

Please outline where this information can be found within the submission (e.g., sections or figure legends), or explain why this information doesn't apply to your submission:

Replicates numbers were based on experience and practical considerations. N numbers and statistical tests are reported in the Materials and Methods section.
Confocal microscopy: Figure 1C & D FRAP experiments and Figure 5B, C and D colocalisation experiments. At least four individual experiments, containing each 2 to 5 bleached cells (each condition contains at least 10 cells and up to 25). No outliers have been excluded.

Replicates

- You should report how often each experiment was performed
- You should include a definition of biological versus technical replication
- The data obtained should be provided and sufficient information should be provided to indicate the number of independent biological and/or technical replicates
- If you encountered any outliers, you should describe how these were handled
- Criteria for exclusion/inclusion of data should be clearly stated
- High-throughput sequence data should be uploaded before submission, with a private link for reviewers provided (these are available from both GEO and ArrayExpress)

Please outline where this information can be found within the submission (e.g., sections or figure legends), or explain why this information doesn't apply to your submission:

Proteomics: Samples were prepared individually three times (biological replicates. These are indicated in reporting figures): Cells were plated, transfected if required, harvested and Co-IP on independent occasions (different days), each time containing all representative samples (i.e. all mutants and wild type control, all time points).
Coomassie stains and Western blotting replicates and uncropped pictures are shown in the supporting material file.

Statistical reporting

- Statistical analysis methods should be described and justified
- Raw data should be presented in figures whenever informative to do so (typically when N per group is less than 10)
- For each experiment, you should identify the statistical tests used, exact values of N, definitions of center, methods of multiple test correction, and dispersion and precision measures (e.g., mean, median, SD, SEM, confidence intervals; and, for the major substantive results, a measure of effect size (e.g., Pearson's r, Cohen's d)
- Report exact p-values wherever possible alongside the summary statistics and 95% confidence intervals. These should be reported for all key questions and not only when the p-value is less than 0.05.

Please outline where this information can be found within the submission (e.g., sections or figure legends), or explain why this information doesn't apply to your submission:

Statistical analysis information can be found at the relevant subsection in Materials and methods.
Proteomics processed data and statistical values can be found in the respective Source Data tables.

(For large datasets, or papers with a very large number of statistical tests, you may upload a single table file with tests, Ns, etc., with reference to sections in the manuscript.)

Group allocation

- Indicate how samples were allocated into experimental groups (in the case of clinical studies, please specify allocation to treatment method); if randomization was used, please also state if restricted randomization was applied
- Indicate if masking was used during group allocation, data collection and/or data analysis

Please outline where this information can be found within the submission (e.g., sections or figure legends), or explain why this information doesn't apply to your submission:

NA

Additional data files ("source data")

- We encourage you to upload relevant additional data files, such as numerical data that are represented as a graph in a figure, or as a summary table
- Where provided, these should be in the most useful format, and they can be uploaded as "Source data" files linked to a main figure or table
- Include model definition files including the full list of parameters used
- Include code used for data analysis (e.g., R, MatLab)
- Avoid stating that data files are "available upon request"

Please indicate the figures or tables for which source data files have been provided:

Figure 2—Source Data 1. Processed mass-spectrometry data for CoIP of H3-eGFP WT and mutants. Used in Figure 2A, B and E.
Figure 2—source data 2. Processed mass-spectrometry data for CoIP of H4-eGFP WT and mutants. Used in Figure 2C, D and F.
Figure 3—Source Data 1. Processed mass-spectrometry data for eGFP-sNASP CoIP native gel bands. Total Precursor Intensity used in Figure 3G.
Figure 5—Source Data 1. mCherry (control), mCherry-Imp4 and mCherry-Imp5 versus H3-eGFP-OMP25 Pearson's correlation coefficients used in Figure 5C.
Figure 5—Source Data 2. eGFP-Imp5 and H3-mCherry-RAPID-release Pearson's correlation coefficients used in Figure 5E.
Figure 5—Source Data 3. H3.1-eGFP-RAPID-release and H3.1-APEX2-eGFP-RAPID-release nuclear localisation used in Figure 5F.
Figure 5—Source Data 4. Processed mass-spectrometry data for APEX2-OMP25 control. Supports Figure 5G.
Figure 5—Source Data 5. Processed mass-spectrometry data for H3.1-APEX2 at 0 and 120 mins after addition of rapamycin. Total Precursor Intensity and P-value used in Figure 5G and 5H.
